# Findings from a novel and scalable community-based HIV testing approach to reduce the time required to complete point-of-care HIV testing in South Africa

**DOI:** 10.1186/s12913-021-07173-x

**Published:** 2021-10-29

**Authors:** Tonderai Mabuto, Geoffrey Setswe, Nolundi Mshweshwe-Pakela, Dave Clark, Sarah Day, Lerato Molobetsi, Jacqueline Pienaar

**Affiliations:** 1 The Aurum Institute NPC, 29 Queens Road, Parktown, 2193 Johannesburg, South Africa; 2 University of South Africa, Preller St, Muckleneuk, Pretoria, South Africa; 3 The University of the Witwatersrand School of Public Health, 60 York Rd, Johannesburg, South Africa; 4 Vanderbilt University, 2201 West End Ave, Nashville, TN USA; 5 The Centre for HIV-AIDS Prevention Studies, 25 St Johns Road, Houghton Estate Johannesburg, South Africa

**Keywords:** South Africa, HIV testing, Community, INSTI

## Abstract

**Background:**

Mobile HIV testing approaches are a key to reaching the global targets of halting the HIV epidemic by 2030. Importantly, the number of clients reached through mobile HIV testing approaches, need to remain high to maintain the cost-effectiveness of these approaches. Advances in rapid in-vitro tests such as INSTI® HIV-1/HIV-2 (INSTI) which uses flow-through technologies, offer opportunities to reduce the HIV testing time to about one minute. Using data from a routine mobile HTS programme which piloted the use of the INSTI point-of-care (POC) test, we sought to estimate the effect of using a faster test on client testing volumes and the number of people identified to be living with HIV, in comparison with standard of care HIV rapid tests.

**Methods:**

In November 2019, one out of four mobile HTS teams operating in Ekurhuleni District (South Africa) was randomly selected to pilot the field use of INSTI-POC test as an HIV screening test (i.e., the intervention team). We compared the median number of clients tested for HIV and the number of HIV-positive clients by the intervention team with another mobile HTS team (matched on performance and area of operation) which used the standard of care (SOC) HIV screening test (i.e., SOC team).

**Results:**

From 19 to 20 December 2019, the intervention team tested 7,403 clients, and the SOC team tested 2,426 clients. The intervention team tested a median of 442 (IQR: 288–522) clients/day; SOC team tested a median of 97 (IQR: 40–187) clients/day (*p*<0.0001). The intervention team tested about 180 more males/day compared to the SOC team, and the median number of adolescents and young adults tested/day by the intervention team were almost four times the number tested by the SOC team. The intervention team identified a higher number of HIV-positive clients compared to the SOC team (142 vs. 88), although the proportion of HIV-positive clients was lower in the intervention team due to the higher number of clients tested.

**Conclusions:**

This pilot programme provides evidence of high performance and high reach, for men and young people through the use of faster HIV rapid tests, by trained lay counsellors in mobile HTS units.

**Supplementary Information:**

The online version contains supplementary material available at 10.1186/s12913-021-07173-x.

## Background

Community-based HIV testing services are a key to reaching the global targets of halting the HIV epidemic by 2030 [1]. In particular, the delivery of HIV testing services (HTS) through mobile units has been shown to be cost-effective and successful in reaching people who are less likely to undergo testing in health care facilities [2–4]. Mobile units provide HTS through outreach teams and fully contained medical mobile units within community settings in order to access underserved or hard to reach populations, such as men, key populations, rural communities, or migrant populations [5]. This includes people who have a low perceived risk of being infected with HIV, are uncomfortable with or unable to frequent available health care facilities, or lack clinical reasons to seek services in health care facilities [6–8].

Although mobile HTS approaches have been shown to be cost-effective, they are not necessarily inexpensive [3]. Compared with facility-based or stand-alone HTS approaches, mobile HTS approaches incur higher costs for capital and recurring expenses as a proportion of totals costs [9]. However, costing studies of community-based HTS approaches in South Africa and similar settings, have shown that the mean cost per HTS client is reduced when client testing volumes are high [3, 9, 10]. As such, the number of clients reached through mobile HTS approaches, needs to remain sufficiently high to maintain the cost-effectiveness of these approaches. However, the turnaround time for obtaining results from current HIV rapid point-of-care tests, means that counsellors are often near capacity with the numbers of clients they can test a day. The standard HIV rapid tests take 10-20 min to produce a result, during which the counsellor and client are occupied, and the service is unavailable for the next client. In South Africa, a negative test is followed by brief post-test counselling, and a positive test is followed by a confirmatory test and post-test counselling [11]. The full process is time consuming with much of the time spent waiting for test results. This constrains the volume of HTS because a single counsellor is limited to testing 2-3 clients per hour and the lack of private spaces in a mobile setup limits the number of counsellors that can provide HTS at any given time [12].

In some mobile HTS settings, onsite HIV self-screening (HIVSS) has been implemented as an approach to increase the reach of HTS [13]. However, despite HIVSS shifting the locus of control to the client, the turnaround time of 15–20 min for test results remains a bottleneck for optimising daily HTS reach. Recent advances in rapid in-vitro tests such as INSTI® HIV-1/HIV-2 (INSTI) which uses faster flow-through technologies (compared with widely available lateral flow-based tests), offer opportunities to reduce the HIV testing time to about one minute of starting the test [14]. The reduced testing times of the INSTI point-of care (POC) test have also been shown to result in high levels of satisfaction by both clients and HTS providers [15, 16]. However, despite the prequalification of the INSTI-POC test by the World Health Organisation (WHO) in 2013, and its transformative potential to increase the volume of HTS in high burden settings; there are few published findings on the implementation outcomes from using INSTI in routine programmes or research settings [15, 17].

Using data from a routine mobile HTS programme which piloted the use of the INSTI-POC test to reduce HIV testing times, we sought to estimate the effect of INSTI-POC on client testing volumes and the number of people identified to be living with HIV, in comparison with standard of care rapid HIV testing approaches.

## Methods

### Programme setting

The mobile HTS programme operated in the Ekurhuleni District (Gauteng Province) of South Africa. Ekurhuleni District is a key district for South Africa’s National Strategic Plan to control the HIV epidemic [18]. In 2019, about 19 % of the estimated 3.7 million people who resided in the district, were living with HIV [19, 20]. The district is characterised by high levels of unemployment and poverty, coupled with increasing numbers of in-migrants who dwell in informal settlements mostly comprising of informal structures not approved for a permanent dwelling [21]. Four mobile HTS teams were deployed across the district in communities, workplaces, commercial shopping areas, and at special events as part of routine service delivery. All counsellors were trained and certified to provide HTS. HIV testing was provided in small private tents/gazebos and in private spaces within the repurposed fully contained medical mobile vans. Further, all HIV testing was offered as an opt-in service requiring informed consent, using finger-prick blood samples, and was accompanied by pre- and post-test counselling in a confidential private setting.

### Piloting use of INSTI-POC as an HIV screening test

 The South Africa HTS guidelines require counsellors to follow a serial testing algorithm, where a first rapid HIV test is performed as a screening test. If the screening test is non-reactive, the client is issued with a HIV-negative result. If the screening test is reactive, the HIV test is repeated with a different rapid HIV test to confirm the result of the screening test.

In November 2019, one out of four mobile HTS teams (hereafter referred to as the intervention team) operating in Ekurhuleni District was randomly selected to pilot the field use of INSTI-POC test as an HIV screening test. Prior to commencing the pilot programme, counsellors in the intervention team received additional three-day training on INTSI-POC tests which covered the principles of the test, conducting the test, interpretation of test results, storage of test kits, and quality control of the testing process. Of the remaining three mobile HTS teams, one team with similar number of counsellors and performance to the intervention team in terms of client testing volumes and the yield of HIV-positive diagnoses (i.e., in the 12-month period prior to study commencement), was selected to serve as the comparison for the intervention team (hereafter referred to as the standard of care (SOC) team). In contrast to the INSTI-POC team, the SOC team used the Abon^TM^ HIV (Abon) rapid diagnostic test, which is the approved standard-of-care HIV screening test in public sector HIV testing programs in South Africa. Both INSTI-POC and Abon tests detect HIV Type 1 and/or Type 2 (HIV-1/HIV-2) and have similar sensitivity and specificity performance characteristics (i.e., >99 % sensitivity and specificity for both tests) [22, 23]. For both the intervention team and SOC team, all screening tests with reactive results were repeated using the First Response^TM^ HIV (First Response) rapid diagnostic test to confirm the screening test result. Therefore, we hypothesise that the INSTI-POC and First Response testing algorithm compared to the Abon and First Response algorithm, had similar theoretical sensitivity and specificity. The Abon test required at least 10 min to read the result, compared with INSTI-POC test which required at least one minute to read the result. All HIV-positive clients were assigned to linkage officers who facilitated linkage to HIV medical care at referral clinics.

### Quality control of test kits

Prior to commencement of the INSTI-POC pilot programme, a batch of INSTI-POC test kits were submitted for post-market surveillance testing at a national reference laboratory. The quality control (QC) process included tests for: (1) known negative and positive samples, (2) analytic sensitivity using a dilution series of reference material, and (3) intra-assay precision testing. Overall, the batch of INSTI-POC kits passed all QC tests. Similar additional QC processes were not conducted for Abon and First Response test kits since they are the approved SOC tests for use in the public sector HIV program with a routine QC process. As per routine standard operating procedures, each counsellor performed weekly internal QC tests on HIV test kits in use and additional QC tests on new shipments of HIV test kits. QC measures were used to assess the stability of the test devices and other reagents used for testing as well as adherence to test standard procedure and requirements. All QC results were recorded and reviewed by team leaders on a weekly basis.

### Data collection

All screening and confirmatory (where applicable) test results were captured by counsellors using an electronic HIV testing register. The HIV testing register included information on the date of HIV test, a unique system-generated client identifier, client’s name, locator details, client’s age, client’s sex, final HIV test result, and a unique counsellor code used to identify the counsellor and the mobile HTS unit in which the client was tested.

### Data analysis

Our analyses had two primary outcomes that served as proxies of HTS performance in the study period, (1) the number of clients tested for HIV and (2) the number of clients identified to be living with HIV (i.e., final HIV test results). To explore the effect of using INSTI-POC on these outcomes, we restricted comparisons to the SOC team on days when both the intervention team and SOC team delivered HTS. Further, we excluded days when one team worked beyond the average 8-hour workday, which occurred when mobile HTS teams were invited to, or hosted special events to provide HTS.

We used the median and interquartile range (IQR) to summarise the number of clients tested per day, and performed nonparametric tests for equality of the median number of clients tested between the two teams, using the Wilcoxon rank sum test [24]. We further compared the median number of clients tested per day among two priority groups for HTS in South Africa: men and adolescents and young adults (15–24 years) [25].

We summarised the final HIV test results (i.e., HIV-negative, or HIV-positive) using number (n) and proportion (%), and further stratified final HIV test results by client’s sex and age group. We explored differences in final HIV test results between the two teams using the Chi-Squared test at 5 % significance level. Data were analysed using STATA ® (Version 16, Stata Corporation, College Station, TX, USA). All data were de-identified for the purposes of this analysis.

## Results

### Comparison of HTS reach between the Intervention and SOC teams

From 19 November 2019 to 20 December 2019 (i.e., study period), both the intervention team and SOC team delivered mobile HTS services for 18 days. On these days, the intervention team tested 7,403 clients compared with 2,426 clients tested by the SOC team. The age and sex distribution of HTS clients was similar between the two teams (Table 1). Both teams mostly reached males, and clients aged 25–49-years. The intervention team tested a median of 442 (IQR: 288–522) clients/day compared with the SOC team which tested a median of 97 (IQR: 40–187) clients/day. Based on 9 counsellors per team, this translated to approximately each counsellor in the intervention team testing about 49 clients/day compared with a counsellor in the SOC team testing about 11 clients/day. The intervention team tested about 180 more males/day compared to the SOC team, and the median number of adolescents and young adults (15-24 years) tested/day by the intervention team (112, IQR: 72–141) were almost four times the number tested by the SOC team (29, IQR: 10–47) (Table 2).

**Table 1:** Characteristics of clients tested by the Intervention and SOC teams

		SOC Team *N* = 2,426		Intervention team *N* = 7,403	
		n	%	n	%
**Sex**	Males	1238	(51.0)	3906	(52.8)
	Females	1188	(48.9)	3497	(47.2)
**Age group (years)**	<15	43	(1.8)	83	(1.1)
	15-24	665	(27.4)	1970	(26.6)
	25-49	1599	(65.9)	5033	(68.0)
	50+	119	(4.9)	317	(4.3)

*SOC* standard of care

**Table 2:** Comparison of client testing volumes between Intervention team and SOC team

Indicator	SOC Team *N* = 2,426	Intervention team *N* = 7,403	*p*-value
Median (IQR) number of clients tested/day	97 (40-187)	442 (288-522)	<0.001
Median (IQR) number of male clients tested/day	41 (22-117)	221 (151-275)	<0.001
Median (IQR) number of clients 15-24yrs tested/day	29 (10-47)	112 (72 – 141)	<0.001

*SOC* standard of care, *IQR* interquartile range

### Final HIV testing outcomes

Over the same number of days, the intervention team identified a total of 142 HIV-positive clients compared with the SOC team which identified 88 HIV-positive clients. Relative to the number of clients tested, the SOC team identified a higher proportion of HIV-positive clients (3.6 %) compared with the intervention team (1.9 %). Also, the HIV-positivity yield was higher among men tested by the SOC team compared with the intervention team (Table 3).

**Table 3:** Comparison of final HIV test outcomes between Intervention team and SOC team

	SOC Team			Intervention team			Chi-square *p*-value
**Category**	Number tested for HIV	HIV-positive diagnosis n (%); 95 % CI		Number tested for HIV	HIV-positive diagnosis n (%); 95 % CI		
**All clients**	2426	88 (3.6)	2.9, 4.4	7403	142 (1.9)	0.2, 2.3	<0.001
**Male clients**	1238	43 (3.5)	2.5, 4.7	3906	65 (1.7)	1.3, 2.1	<0.001
**15-24 years clients**	665	7 (1.1)	0.4, 2.2	1970	12(0.6)	0.3, 1.0	0.24

*SOC* standard of care, *IQR* interquartile range, *CI* confidence interval

## Discussion

Identifying ways to increase the reach of HTS is essential to reach the goals set globally for reducing the number of new infections and AIDS-related deaths. In this retrospective analysis of data from a routine mobile HTS programme, we identified the transformative potential of a faster HIV screening test to increase the reach of mobile HTS services. The large differences in the number of HTS clients reached between the intervention team and SOC team point to the potential benefit of faster HIV rapid testing technologies to increasing the productivity of healthcare workers providing HTS.

Achieving the balance in increasing the volume of HTS, while maintaining the quality of HIV test results is essential for increasing the number of people aware of their HIV status without causing unintended harm due to poor quality of HIV testing procedures. This is of particular concern in South Africa where about 10 million HIV tests are performed annually using rapid HIV diagnostic tests [19], and up to about 2.4 % and 8.9 % of the test results are false positive and false negative results, respectively [26, 27]. While the country has intensified efforts of training and quality assurance programmes to improve the quality control and adherence to testing algorithms [28], little has been documented about the malpractices by HTS providers in prematurely reading test results before the recommended minimum time [29]. It is plausible that poor fidelity to result waiting times is a result of HTS providers’ efforts to reduce patient waiting times or an attempt to meet daily performance targets. concordance test. Our findings of high HTS reach from the intervention team, suggest that with faster HIV screening tests, HTS providers may be able to increase the number of clients tested without the need to prematurely read the test results in an effort to save time.

Over the past decade, the reach of HTS to adolescents and young adults and men in South Africa has been suboptimal to meet the country’s goal of reducing the proportion of people with undiagnosed HIV infection to at least 5% [25, 30]. While community-based HTS has been shown to reach these hard-to-reach population groups [8], the number needed to test to identify people living with HIV is set to increase as the proportion of people aware of their HIV status increases. Our findings show that the intervention team reached more than triple the number of clients compared with the SOC team. We hypothesise that shorter queues and faster service times at the intervention team’s mobile HTS sites may have increased demand of services by reducing the perceived opportunity costs of undergoing HTS or reduced the potential of loss in privacy by being seen in HIV testing queues [31, 32]. These are particularly important factors for mobile HTS units that offer services to people in public places, and to people who may have competing priorities at the time they are invited for HTS (e.g., shopping or attending an event). With the advent and high uptake of HIV self-screening strategies in South Africa, faster HIV self-screening test may also increase the throughput of community-based HTS [13, 33, 34].

Our findings of high reach and low HIV-positivity yield from the intervention team, compared with low reach and marginally higher HIV-positivity yield from the SOC team, contribute to ongoing discussions on how to best measure the cost efficiency of HTS programmes (i.e., absolute numbers vs. yield) [35]. Achieving universal health coverage for people living with HIV, requires identifying more people unaware of their HIV diagnosis, and our findings show that this can be achieved through faster rapid HIV diagnostic tests. Further, achieving HIV epidemic control also requires identifying HIV-negative persons and linking them to HIV prevention programmes to reduce the number of new HIV infections, which is a frequently overlooked aspect in evaluating the cost-effectiveness of mobile HTS approaches.

Our study highlights the field performance of counsellors using a fast HIV screening test and the potential benefit in increasing HTS reach, but it is not without limitations. Our comparison of HTS reach between the Intervention and SOC teams, was conducted within a pragmatic setting where teams identified target areas for HTS, as per routine practice. While it is plausible that the teams served areas with different HTS demand, it is noteworthy that these teams were selected because of similar performance over a period of 12 months, and the team composition did not change during the study period. Therefore, to a large extent, the observed differences in HTS reach between the two teams may be attributed to the potential influence of using a faster HIV screening test. One of the goals of HTS is to ensure that people with an HIV-positive diagnosis are linked to HIV care services. Data on linkage-to-care outcomes from clinic records could not be readily linked to unique identifiers in the de-identified data set and was excluded from this analysis. Further, we were unable to quantify the number of clients who were previously unaware of their HIV-positive. However, emerging evidence suggests that clients who have never entered care or those who have disengaged from care, use HTS as a gateway for first entry or re-entry into HIV care and treatment services [36, 37]. Lastly, we report findings from a routine mobile HTS programme in an urban high HIV-burden district, using data collected over an 18-day period. In this regard, our findings need to be interpreted cautiously beyond the program’s implementation context.

## Conclusions

This pilot programme provides preliminary evidence of high HTS reach through the use of a faster HIV screening test by trained lay counsellors in mobile HTS units. As South Africa continues its investments in HIV prevention and treatment programmes, innovations such as INSTI-POC that reduce the time spent utilising HIV services will be key to success. We hypothesize that the gains in productivity outweigh the increase in test kit costs, but this needs further study. Our work sets a platform for further research exploring implementation and health economics outcomes to assess the benefits of faster HIV screening in community-based HTS models.

## Supplementary information

**Additional file 1.**

## Data Availability

The datasets generated and/or analysed during the current study are not publicly available as they pertain to routine programme data but are available from the corresponding author on reasonable request and upon receipt of relevant approvals by local authorities in South Africa.

## Abbreviations

CI: Confidence Interval
HTS: HIV testing services
IQR: Interquartile range
POC: Point-of-care
QC: Quality Control
SOC: Standard of care
WHO: World Health Organisation
