# Correction to: Findings from a novel and scalable community-based HIV testing approach to reduce the time required to complete point-of-care HIV testing in South Africa

**DOI:** 10.1186/s12913-021-07271-w

**Published:** 2021-11-22

**Authors:** Tonderai Mabuto, Geoffrey Setswe, Nolundi Mshweshwe-Pakela, Dave Clark, Sarah Day, Lerato Molobetsi, Jacqueline Pienaar

**Affiliations:** 1 The Aurum Institute NPC, 29 Queens Road, Parktown, Johannesburg, 2193 South Africa; 2 University of South Africa, Preller St, Muckleneuk, Pretoria, South Africa; 3 The University of the Witwatersrand School of Public Health, 60 York Rd, Johannesburg, South Africa; 4 Vanderbilt University, 2201 West End Ave, Nashville, TN USA; 5 The Centre for HIV-AIDS Prevention Studies, 25 St Johns Road, Houghton Estate, Johannesburg, South Africa

**Correction to: BMC Health Serv Res 21, 1176 (2021)**

**https://doi.org/10.1186/s12913-021-07173-x**

Following publication of the original article [1], an error was identified in the Results section of the Abstract.

The updated Results is given below and the changes have been highlighted in **bold typeface**.

From 19 **November 2019** to 20 December 2019, the intervention team tested 7,403 clients, and the SOC team tested 2,426 clients.

The original article [1] has been corrected.
