# Supplementary material for: Findings from a novel and scalable community-based HIV testing approach to reduce the time required to complete point-of-care HIV testing in South Africa
Source: BMC Health Serv Res. 2021 Oct 29;21:1176. doi: 10.1186/s12913-021-07173-x (PMC8555215; doi:10.1186/s12913-021-07173-x)
Supplement: Supplementary file 1 — Additional file 1. [file 12913_2021_7173_MOESM1_ESM.pdf]

Additional Files

Additional File 1: INSTI-POC Post-marketing Surveillance Test Report

File Format – PDF

Title of Data - INSTI-POC Post-marketing Surveillance Test Results

Description of data – Prior to commencement of the INSTI-POC pilot programme, a batch of INSTI-POC test kits were submitted for post-market surveillance testing at a national reference laboratory. The QC process included tests for: (1) known negative and positive samples, (2) analytic sensitivity using a dilution series of reference material, and (3) intra-assay precision testing. Overall, the batch of INSTI-POC kits passed all QC tests

NATIONAL INSTITUTE FOR COMMUNICABLE DISEASES
Division in the National Health Laboratory Service

POST MARKETING SURVEILLANCE TEST REPORT

Date of Test Report: 17 September 2019

Supplier Name	**The Aurum Institute**
Manufacturer Name	**BioLytical Laboratories**
Name of Test Kit	**Insti HIV-1/HIV-2 Antibody Test**
Version Number of Package Insert	**51-1097L**
Kit Lot Number	**801010190142**
Kit Expiry Date	**15/05/2020**
Date of Receipt	**12/09/2019**
Type of Testing	**Post Market Surveillance**
Date of Testing	**16/09/2019 – 17/09/2019**

**Results meet the specifications according to NICD verification criteria**	**Yes ☒ No ☐**

COMMENTS:

AJ PUREN
Signature of Deputy Director/Nominated Deputy – Centre for HIV and STI

17th September 2019
Date

In the event of a dispute concerning this document, the electronic version stored on Q-Pulse will be deemed to be the correct version

National Health Laboratory Service- All rights reserved

NATIONAL INSTITUTE FOR COMMUNICABLE DISEASES
Division in the National Health Laboratory Service

DETAILS OF TESTING

METHOD AND MATERIAL USED

Test Procedure	The test performance strictly follows the current instructions for use as provided by the manufacturer
Incubation Period	Time to read per instructions of use
Samples Tested	South African National Blood services samples (SANBS) (clarified and characterised serum samples)<br><br>Dilution series – 5 independent samples from SANBS diluted to end point ((clarified and characterised serum samples)<br><br>Whole blood clinical samples

OPERATORS

	Name and Surname	Date
Technical Performance (Operator 1)	Sara Hloma<br>Thabo Phokela	16/09/2019 – 17/09/2019<br>17/09/2019
Technical Performance (Operator 2)	Sara Hloma<br>Thabo Phokela<br>Candice Subramunian	17/09/2019<br>16/09/2019-17/09/2019<br>16/09/2019
Technical Performance Reviewed by:	Deirdre Greyling	17/09//2019

SUMMARY OF TEST RESULTS

1. **INTERNAL QUALITY CONTROL RESULTS (IQC)**

	IQC Lot Number and Expiry Date	CONTROL LINE RESULT	TEST LINE RESULT	EXPECTED RESULTS	CRITERIA MET YES/NO
Negative IQC	Lot: 010219N<br>Exp: 02/2020	Positive	Negative	Negative	Yes
Positive IQC	Lot: 010219P<br>Exp: 02/2020	Positive	Positive	Positive	Yes

National Health Laboratory Service- All rights reserved

NATIONAL INSTITUTE FOR COMMUNICABLE DISEASES
Division in the National Health Laboratory Service

2. ANALYTICAL SENSITIVITY TESTING USING NICD DILUTION SERIES REFERENCE MATERIAL

DILUTION351	CONTROL LINE RESULT	TEST LINE RESULT	EXPECTED RESULTS	CRITERIA MET YES/NO
1:5	Positive	Positive		
1:10	Positive	Positive		
1:20	Positive	Positive		
1:40	Positive	Positive		
1:80	Positive	Positive		
1:160	Positive	Negative		
1:320	Positive	Negative		
1:640	Positive	Negative		
1:1280	Positive	Negative		
1:2560	Positive	Negative		
1:5120	Positive	Negative		
1:10240	Positive	Negative		
1:20480	Positive	Negative		
1:40960	Positive	Negative		
1:81920	Positive	Negative		

Not Scored

In the event of a dispute concerning this document, the electronic version stored on Q-Pulse will be deemed to be the correct version

National Health Laboratory Service- All rights reserved

NATIONAL INSTITUTE FOR COMMUNICABLE DISEASES
Division in the National Health Laboratory Service

DILUTION380	CONTROL LINE RESULT	TEST LINE RESULT	EXPECTED RESULTS	CRITERIA MET YES/NO
1:5	Positive	Positive		
1:10	Positive	Positive		
1:20	Positive	Positive		
1:40	Positive	Positive		
1:80	Positive	Positive		
1:160	Positive	Positive		
1:320	Positive	Positive		
1:640	Positive	Negative		
1:1280	Positive	Negative		
1:2560	Positive	Negative		
1:5120	Positive	Negative		
1:10240	Positive	Negative		
1:20480	Positive	Negative		

Not Scored

In the event of a dispute concerning this document, the electronic version stored on Q-Pulse will be deemed to be the correct version

National Health Laboratory Service- All rights reserved

DILUTION435	CONTROL LINE RESULT	TEST LINE RESULT	EXPECTED RESULTS	CRITERIA MET YES/NO
1:5	Positive	Positive		
1:10	Positive	Positive		
1:20	Positive	Positive		
1:40	Positive	Positive		
1:80	Positive	Positive		
1:160	Positive	Positive		
1:320	Positive	Positive		
1:640	Positive	Positive		
1:1280	Positive	Negative		
1:2560	Positive	Negative		
1:5120	Positive	Negative		

Not Scored

In the event of a dispute concerning this document, the electronic version stored on Q-Pulse will be deemed to be the correct version

National Health Laboratory Service- All rights reserved

NATIONAL INSTITUTE FOR COMMUNICABLE DISEASES
Division in the National Health Laboratory Service

DILUTION480	CONTROL LINE RESULT	TEST LINE RESULT	EXPECTED RESULTS	CRITERIA MET YES/NO
1:5	Positive	Positive		
1:10	Positive	Positive		
1:20	Positive	Positive		
1:40	Positive	Positive		
1:80	Positive	Weak Positive		
1:160	Positive	Negative		
1:320	Positive	Negative		
1:640	Positive	Negative		
1:1280	Positive	Negative		
1:2560	Positive	Negative		
1:5120	Positive	Negative		
1:10240	Positive	Negative		
1:20480	Positive	Negative		

Not Scored

In the event of a dispute concerning this document, the electronic version stored on Q-Pulse will be deemed to be the correct version

National Health Laboratory Service- All rights reserved

NATIONAL INSTITUTE FOR COMMUNICABLE DISEASES
Division in the National Health Laboratory Service

DILUTION 492	CONTROL LINE RESULT	TEST LINE RESULT	EXPECTED RESULTS	CRITERIA MET YES/NO
1:5	Positive	Positive		
1:10	Positive	Positive		
1:20	Positive	Positive		
1:40	Positive	Positive		
1:80	Positive	Positive		
1:160	Positive	Positive		
1:320	Positive	Positive		
1:640	Positive	Weak Positive		
1:1280	Positive	Negative		
1:2560	Positive	Negative		
1:5120	Positive	Negative		

Not Scored

Agreement*:

Agreement meets criteria

YES (>/=90%)	**Not Scored**
NO (<90%)	

*** Agreement refers to comparison of dilution series on initial batch testing for HIV Rapid Tests and current batch for post marketing surveillance**

In the event of a dispute concerning this document, the electronic version stored on Q-Pulse will be deemed to be the correct version

National Health Laboratory Service- All rights reserved

NATIONAL INSTITUTE FOR COMMUNICABLE DISEASES
Division in the National Health Laboratory Service

3. INTRA-ASSAY PRECISION TESTING USING LAST POSITIVE IN THE DILUTION SERIES PANEL

DILUTION	CONTROL LINE RESULT	TEST LINE RESULT	EXPECTED RESULTS	CRITERIA MET YES/NO
459 1:320	Positive	Positive	Positive	Yes
459 1:320	Positive	Positive	Positive	Yes
459 1:320	Positive	Positive	Positive	Yes
459 1:320	Positive	Positive	Positive	Yes
459 1:320	Positive	Positive	Positive	Yes

Agreement*:

All bands have equal intensity across all specimens during the precision testing

Agreement meets criteria

YES (>/=80%)	**Yes = 100%**
NO (<80%)	

In the event of a dispute concerning this document, the electronic version stored on Q-Pulse will be deemed to be the correct version

National Health Laboratory Service- All rights reserved

NATIONAL INSTITUTE FOR COMMUNICABLE DISEASES
Division in the National Health Laboratory Service

4.ANALYTICAL SENSITIVITY TESTINGWITH NICD RECENTLY INFECTED HIV REFERENCE MATERIAL

SAMPLE NUMBER	CONTROL LINE RESULT	TEST LINE RESULT	EXPECTED RESULTS	CRITERIA MET YES/NO
REC001	Positive	Positive	Positive	Yes
REC002	Positive	Positive	Positive	Yes
REC003	Positive	Positive	Positive	Yes
REC004	Positive	Positive	Positive	Yes
REC005	Positive	Positive	Positive	Yes
REC006	Positive	Positive	Positive	Yes
REC007	Positive	Positive	Positive	Yes
REC008	Positive	Positive	Positive	Yes
REC009	Positive	Positive	Positive	Yes
REC010	Positive	Positive	Positive	Yes
REC011	Positive	Positive	Positive	Yes
REC012	Positive	Positive	Positive	Yes
REC013	Positive	Positive	Positive	Yes
REC014	Positive	Positive	Positive	Yes
REC015	Positive	Positive	Positive	Yes

Agreement*:

Agreement meets criteria

YES (>/=90%)	**Yes = 100%**
NO (<90%)	

National Health Laboratory Service- All rights reserved

***Agreement refers to comparison of recently infected samples on initial batch testing and current batch for post marketing surveillance**

NATIONAL INSTITUTE FOR COMMUNICABLE DISEASES
Division in the National Health Laboratory Service

4. ANALYTICAL SENSITIVITY TESTINGWITH LOW SERUM/CUT-OFF RATIO HIV REFERENCE MATERIAL

SAMPLE NUMBER	CONTROL LINE RESULT	TEST LINE RESULT	EXPECTED RESULTS	CRITERIA MET YES/NO
LOW001	Positive	Positive	Positive	Yes
LOW002	Positive	Positive	Positive	Yes
LOW003	Positive	Positive	Positive	Yes
LOW004	Positive	Positive	Positive	Yes
LOW005	Positive	Weak Positive	Positive	Yes

Agreement*:

Agreement meets criteria

YES (>/=80%)	**Yes = 100%**
NO (<80%)	

***Agreement refers to comparison of low S/CO samples on initial batch testing and current batch for post marketing surveillance**

In the event of a dispute concerning this document, the electronic version stored on Q-Pulse will be deemed to be the correct version

National Health Laboratory Service- All rights reserved

NATIONAL INSTITUTE FOR COMMUNICABLE DISEASES
Division in the National Health Laboratory Service

5. ANALYTICAL SENSITIVITY WITH NICD ANTI-HIV-1 AND ANTI-HIV-2 REFERENCE MATERIAL

SAMPLE NUMBER	CONTROL LINE RESULT	TEST LINE RESULT	EXPECTED RESULTS	CRITERIA MET YES/NO
PMS027	Positive	Negative	Negative	Yes
PMS028	Positive	Negative	Negative	Yes
PMS029	Positive	Positive	Positive	Yes
PMS030	Positive	Negative	Negative	Yes
PMS031	Positive	Positive	Positive	Yes
PMS032	Positive	Positive	Positive	Yes
PMS033	Positive	Positive	Positive	Yes
PMS034	Positive	Negative	Negative	Yes
PMS035	Positive	Negative	Negative	Yes
PMS036	Positive	Negative	Negative	Yes
PMS037	Positive	Positive	Positive	Yes
PMS038	Positive	Negative	Negative	Yes
PMS039	Positive	Negative	Negative	Yes
PMS040	Positive	Positive	Positive	Yes
PMS041	Positive	Positive	Positive	Yes
PMS042	Positive	Positive	Positive	Yes
PMS043	Positive	Positive	Positive	Yes
PMS044	Positive	Negative	Negative	Yes
PMS045	Positive	Negative	Negative	Yes
PMS046	Positive	Positive	Positive	Yes
PMS047	Positive	Negative	Negative	Yes
PMS048	Positive	Positive	Positive	Yes
PMS049	Positive	Positive	Positive	Yes
PMS050	Positive	Negative	Negative	Yes

In the event of a dispute concerning this document, the electronic version stored on Q-Pulse will be deemed to be the correct version

National Health Laboratory Service- All rights reserved

NATIONAL INSTITUTE FOR COMMUNICABLE DISEASES
Division in the National Health Laboratory Service

Agreement*:

Agreement meets criteria

YES (>/=90%)	**Yes = 100%**
NO (<90%)	

*** Agreement refers to comparison of low S/CO samples on initial batch testing and current batch for post marketing surveillance**

6. CLINICALSAMPLES USING EDTA WHOLE BLOOD

SAMPLE NUMBER	CONTROL LINE RESULT	CONTROL LINE COLOUR	TEST LINE RESULT	EXPECTED RESULTS	CRITERIA MET YES/NO
**LC00964985**	Positive	Positive	Negative	Negative	Yes
**LC00965018**	Positive	Positive	Negative	Negative	Yes
**LC00965011**	Positive	Positive	Negative	Negative	Yes
**LC00964959**	Positive	Positive	Negative	Negative	Yes
**LC00965004**	Positive	Positive	Negative	Negative	Yes
**LC00965002**	Positive	Positive	Positive	Positive	Yes
**LC00964996**	Positive	Positive	Negative	Negative	Yes
**LC00964951**	Positive	Positive	Positive	Positive	Yes
**LC00965022**	Positive	Positive	Negative	Negative	Yes
**LC00965021**	Positive	Positive	Positive	Positive	Yes

Footnotes:
Control Line Result: Minutes from Sample Application until the Control Line is Coloured
Control Line Colour: Record control line colour: POS, WPOS or NEG
Record under the comments section whether there is interference/background on the test devices

In the event of a dispute concerning this document, the electronic version stored on Q-Pulse will be deemed to be the correct version

National Health Laboratory Service- All rights reserved
